# Supplementary material for: tRNA regulation and amino acid usage bias reflect a coordinated metabolic adaptation in Plasmodium falciparum
Source: iScience. 2024 Oct 12;27(11):111167. doi: 10.1016/j.isci.2024.111167 (PMC11544085; doi:10.1016/j.isci.2024.111167)
Supplement: Document S1. Figures S1–S8 [file mmc1.pdf]

iScience, Volume *27*

Supplemental information

tRNA regulation and amino acid usage bias reflect a coordinated metabolic adaptation in *Plasmodium falciparum*

Qian Li, Leonie Vetter, Ylva Veith, Elena Christ, Ákos Végvári, Cagla Sahin, Ulf Ribacke, Mats Wahlgren, Johan Ankarklev, Ola Larsson, and Sherwin Chun-Leung Chan

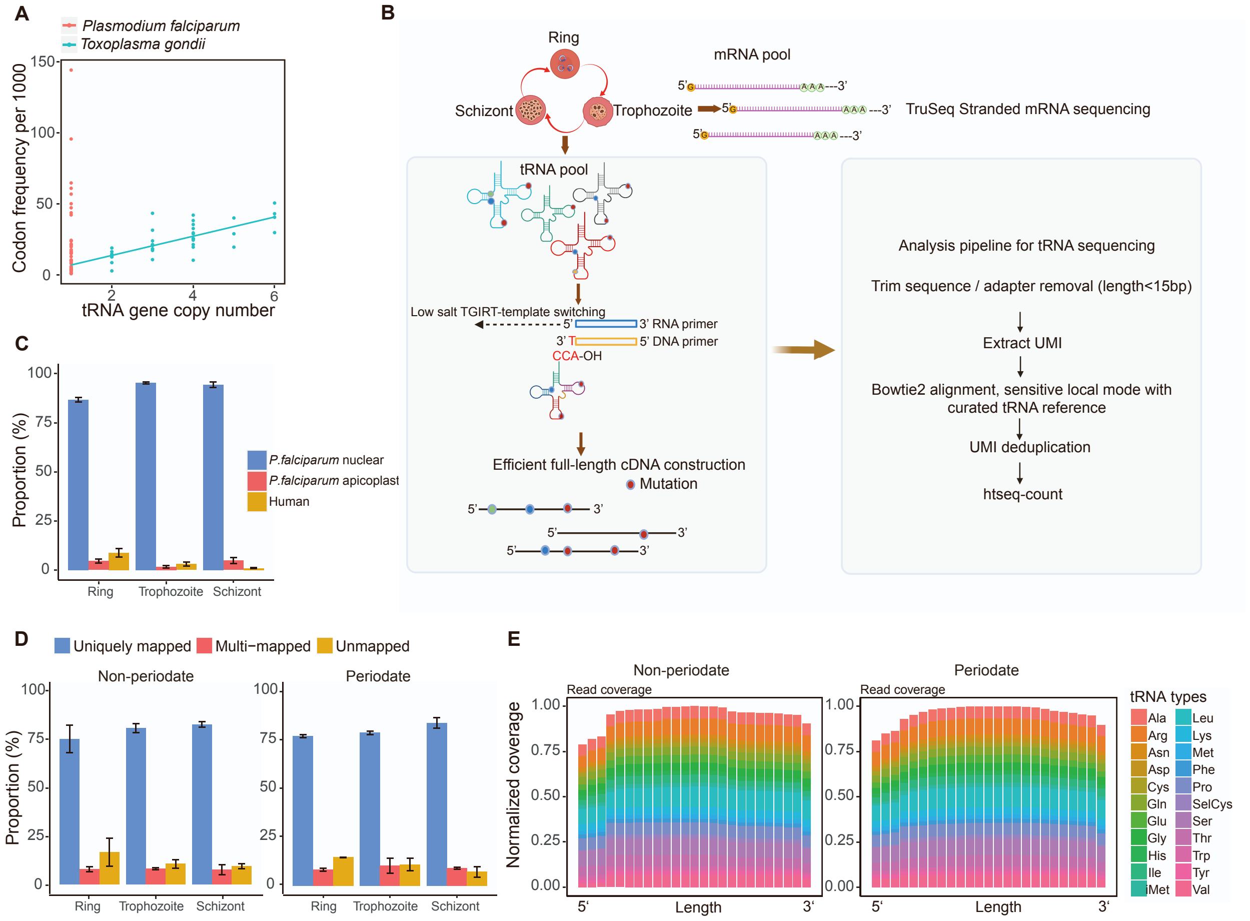

Figure S1. tRNAome analysis of *P. falciparum*, related to Figure 2

(A) Relationship between tRNA gene copy numbers and codon usage in *T. gondii* and *P. falciparum*. **(B)** Left panel: Schematic showing tRNA sequence library generation using TGIRT. A DNA/RNA duplex with a T overhang complementary to the terminal adenosine of mature tRNAs was used for the template-switching reaction. The 3' adapter was ligated after the cDNA was synthesized. Right panel: Strategy for read processing of the tRNA sequencing libraries. **(C)** The origin of all reads mapped to tRNAs throughout the IDC. Error bars represent the standard deviation of N=3 biological replicates. **(D)** Bowtie2 alignment-statistics for tRNA-sequencing data generated from non-periodate (untreated, N=3) and periodate-treated (N=3) libraries. Error bars represent standard deviation from N=3 biological replicates. **(E)** Representative scaled 5'-to-3' sequence coverage plots spanning all nuclear tRNAs. The full-length tRNA was divided into 25 bins with each bin representing 4% of the tRNA length, as shown on the x-axis. The Y-axis value for each tRNA was normalized to the bin with the maximum coverage for each respective tRNA. Left panel: samples without periodate treatment. Right panel: sample treated with periodate. For (C) and (D): data are represented as mean +/- standard deviation of n=3 for each IDC stage.

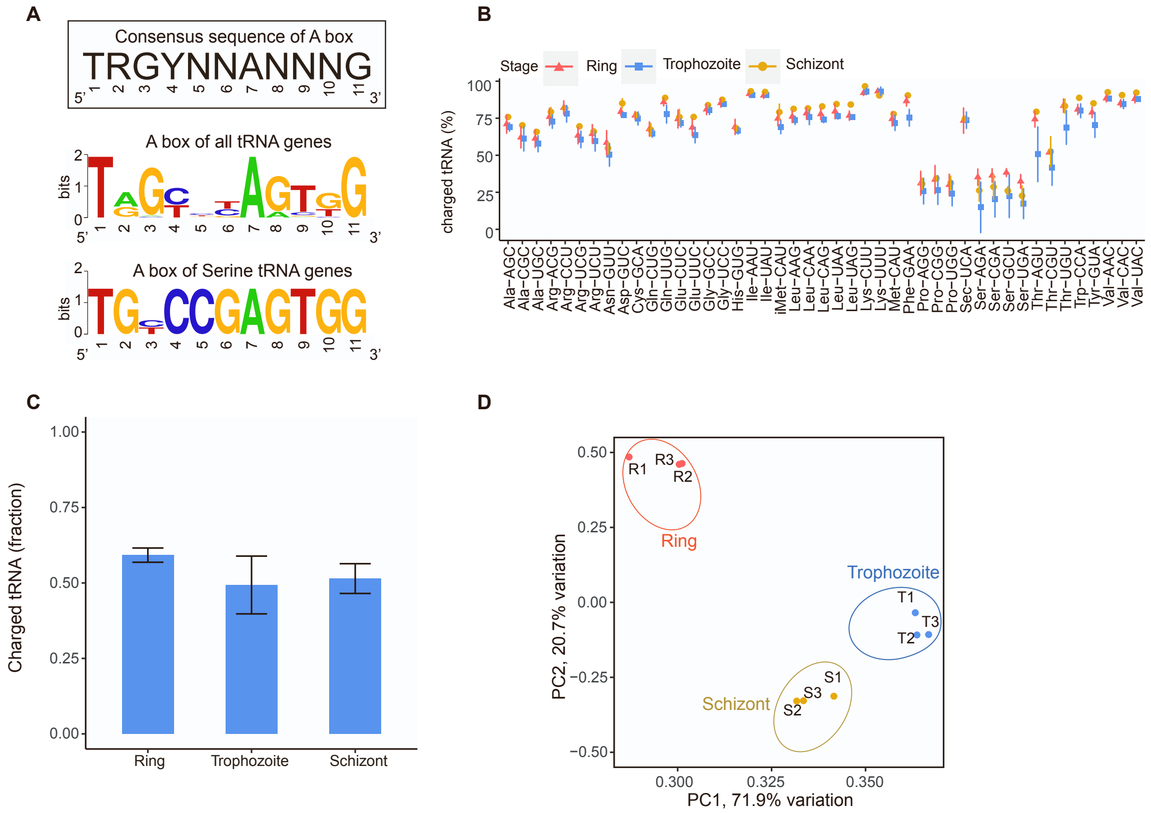

Figure S2. Characteristics of the anticodon and codon pools, related to Figure 2

(A) Motif logo of the A-box in all, except Ser-tRNAs, *P. falciparum* tRNAs (upper), and of all Ser-tRNAs (lower). With a G-to-Y substitution at the third position, the A-box of Ser-tRNAs diverge from the eukaryotic consensus (boxed). **(B,C)** tRNA charging throughout the IDC. Relative charge of (**B**) individual tRNA and **(C)** total tRNA throughout the IDC. Charged tRNAs are represented by reads with 3' -CCA ends after periodate oxidation and beta-elimination reactions. Error bars represent the standard deviation of N=3 biological replicates for Ring and Trophozoite, Schizont: N=2. All apicoplast tRNAs are excluded. **(D)** Principal component analysis of the coding transcriptomes throughout the IDC. For (B) and (C): data are represented as mean +/- standard deviation of n=3 for each IDC stage.

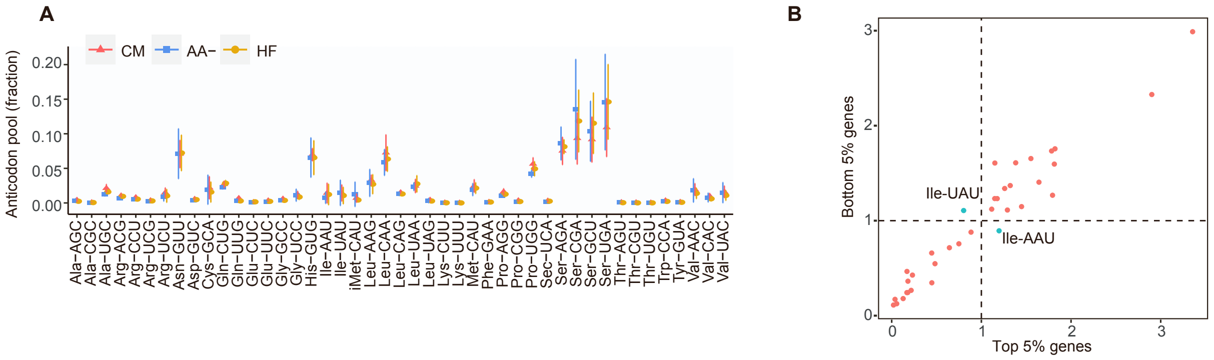

Figure S3. Limited changes in the tRNAome after acute amino acid depletion, related to Figure 3

(A) Changes in tRNA composition after 6 hours of treatment in late stage parasite 32-36 h.p.i. (CM: complete medium; AA-: AA starvation; and HF: halofuginone (70 nM and AA starvation). Error bars represent the SD of N=3 biological replicates. **(B)** The relative isoacceptor usage of the 5% most highly expressed coding genes (x-axis, n=258) and the 5% least expressed coding genes (y-axis, n=258). Relative isoacceptor usage for each tRNA was calculated as the fraction of all codons encoding a specific amino acid that is decoded by an isoacceptor relative to the fraction of all isoacceptors for a specific amino acid that are equally used. The most highly expressed genes preferred Ile-AAU, while lowly expressed genes preferred Ile-UAU. For (A): data are represented as mean +/- standard deviation of n=3 for each IDC stage.

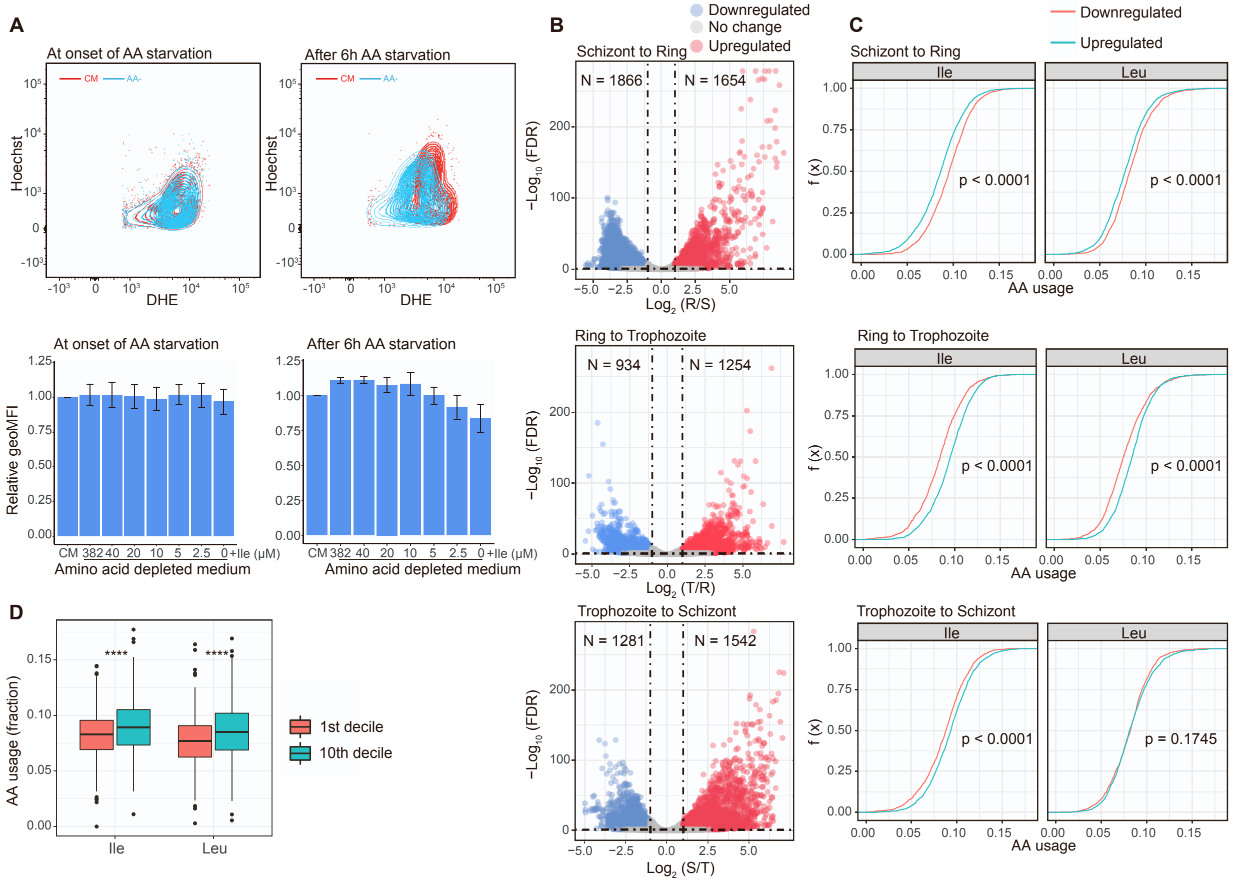

Figure S4. Stage progression is isoleucine sensitive and is delayed by acute amino acid deprivation, related to Figure 3

(A) FACS plots showing the fluorescence intensity of dihydroethidium (DHE) and Hoechst DNA staining in parasites (upper left) at the beginning and (upper right) after 6 hours of AA starvation (AA-) compared to parasites grown in complete medium (CM). Both DHE and Hoechst stainings were reduced in AA- parasites after 6 hours. The geometric mean of the Hoechst staining intensity in parasites (lower left) at the beginning and (lower right) after 6 hours of AA starvation relative to that in CM parasites is shown. Supplementing isoleucine to AA- parasites rescued the reduction in DNA content in a dose-dependent manner. Error bars represent the SD for N=3 biological replicates. **(B)** DESeq2 differential gene expression analyses of the transcriptomes during stage progression from (upper) the schizont-to-ring stage, (middle) the ring-to-trophozoite stage, and (below) the trophozoite-to-schizont stage. The horizontal and vertical dotted lines show the applied adjusted p-value (<0.05) and fold change (2-fold) thresholds, respectively. N=3 biological replicates. **(C)** Empirical cumulative distribution function (CDF) plot of isoleucine and leucine demand in up- (cyan) and down-regulated (red) genes during stage progression. The p-values of Mann–Whitney U tests are indicated. **(D)** Isoleucine and leucine usage in the 10% of genes with the most variable expression (the 10th decile) and the 10% of genes with the least variable expression (1st decile) throughout the IDC. Variability was defined by the coefficient of variation in expression (CV of TPM) throughout the IDC. For (A): data are represented as mean +/- SEM

of n=3 for each IDC stage. For (D): the box plots show the median and the interquartile range, n=526 for both the 1st and 10th decile groups.

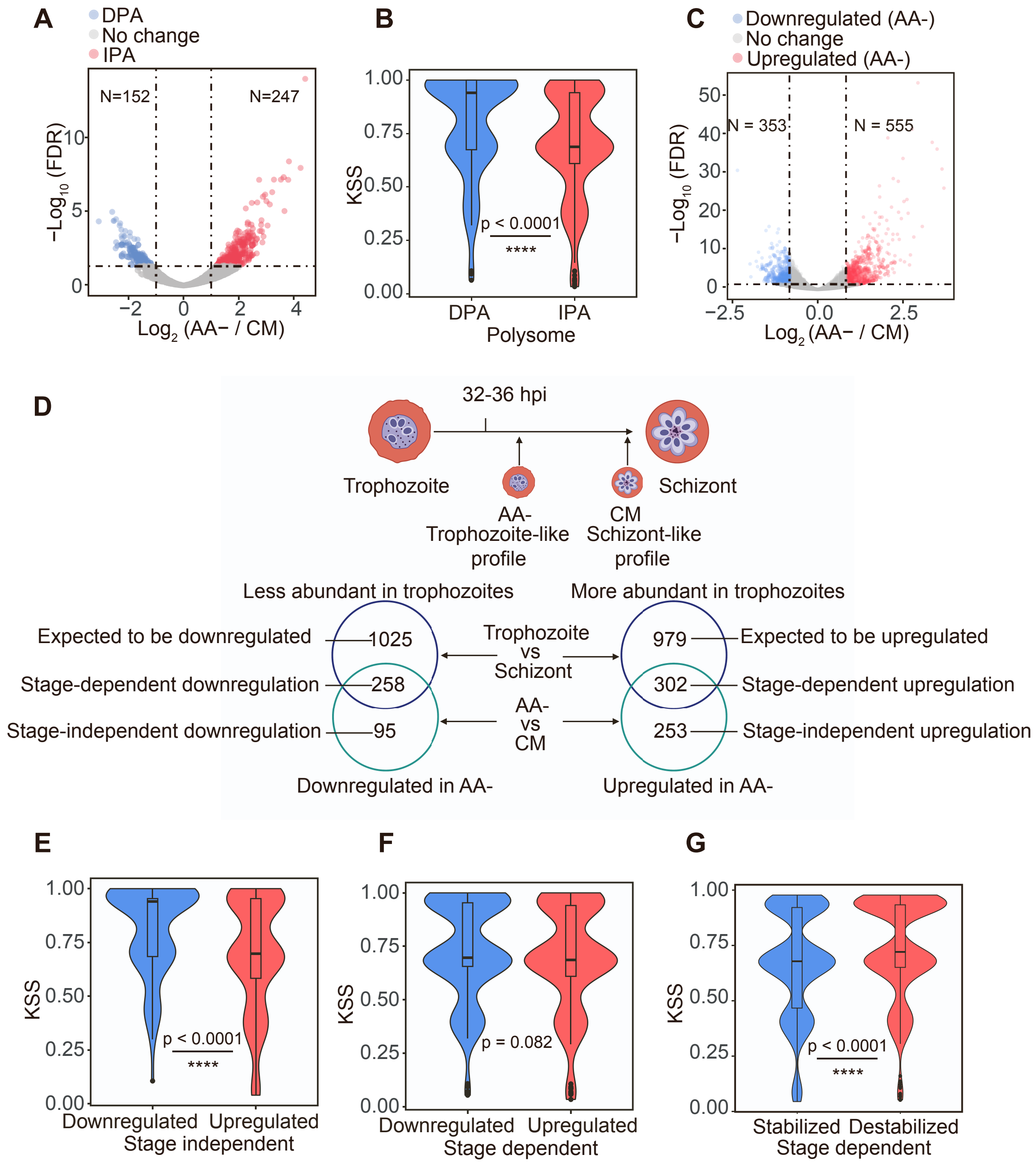

Figure S5. Increased ribosome loading stabilizes transcripts and reprograms the transcriptome, related to Figure 4

(A) DESeq2 differential gene expression analysis for polysome-associated mRNA after AA starvation. The horizontal and vertical dotted lines show applied adjusted p-value (<0.05) and fold changes (2-fold) cutoffs, respectively. **(B)** Kozak similarity scores (KSS, using ribosomal genes as reference) in transcripts with increased polysome association (IPA) or decreased polysome association (DPA) after amino acid depletion. Transcripts with increased polysome association

had lower scores, indicating an initiation context less similar to that of the highly translated ribosome encoding mRNAs. The p-value from a Mann– Whitney U test is indicated. **(C)** DESeq2 differential gene expression analysis of the transcriptome after AA starvation. The horizontal and vertical dotted lines show applied adjusted p-value (<0.05) and fold change (2-fold) cutoffs, respectively. N=3 biological replicates. **(D)** An overview of the strategy used to categorize stage-dependent and stage-independent changes in the transcriptome. The transcriptome of the AA-starved parasites (AA-) was expected to most closely resemble a trophozoite-like transcriptional profile due to delayed stage progression (upper). Transcripts that were more abundant in trophozoites were expected to be upregulated in AA- parasites and vice versa, as illustrated in the Venn diagram. **(E,F)** The KSS of stage-independent **(E)** and stage-dependent **(F)** up- and downregulated genes. Only stage-independent changes show a disparity in KSS between the up- and downregulated genes similar to that in the polysome fractions, supporting a positive feedback loop. The p-values from Mann–Whitney U tests are indicated. **(G)** The KSS of transcripts that remained stable after AA starvation but were expected to be downregulated (and therefore stabilized) or upregulated (and therefore destabilized) according to the categorization shown in (**d**). Statistical significance was assessed using Mann-Whitney U tests in (**E-G**). Resulting p-values are indicated. For (B), (E), (F) and (G): the violin plots show the median, the interquartile range and the distribution of the data.

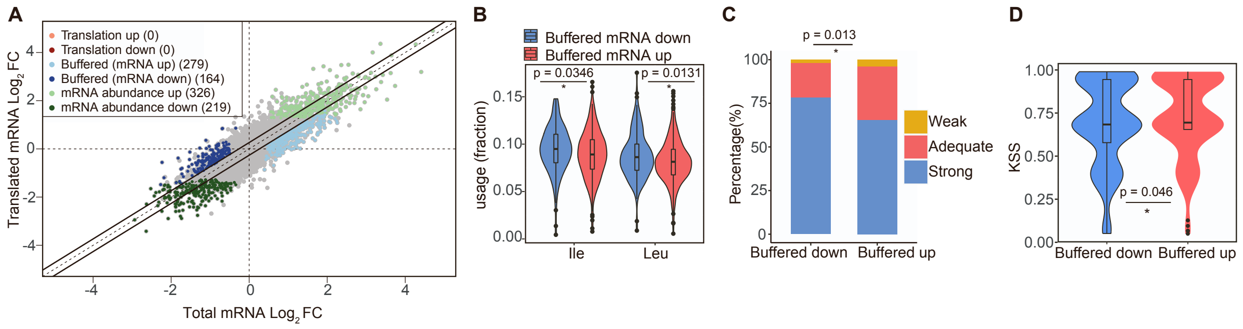

Figure S6. Anota2seq analysis reveals an abundance of translationally "buffered" transcripts, related to Figure 4

(A) Anota2seq analysis of the polysome-profiling dataset using rlog normalization with default parameters. Anota2seq performs analysis of multiple gene expression modes. Genes classified as buffered (mRNA down) correspond to those with a relative increase in ribosome loading but with no transcript-stabilizing effect. N=3 biological replicates. **(B-D)** The differences in isoleucine and leucine usage **(B)**, the Kozak context **(C)**, and KSS **(D)** in "buffered (mRNA up)" and "buffered (mRNA down)" transcripts.

Kozak context: strong, RnnATGR; adequate, RnnATGY/YnnATGR; and weak, YnnATGY (ATG= start codon).

Statistical significance was assessed using unpaired Student's t-test in **(B,D)**; and Fisher's exact test for the Kozak context analysis in **(C)**. Resulting p-values are indicated. For (B) and (D): the violin plots show the median, the interquartile range and the distribution of the data.

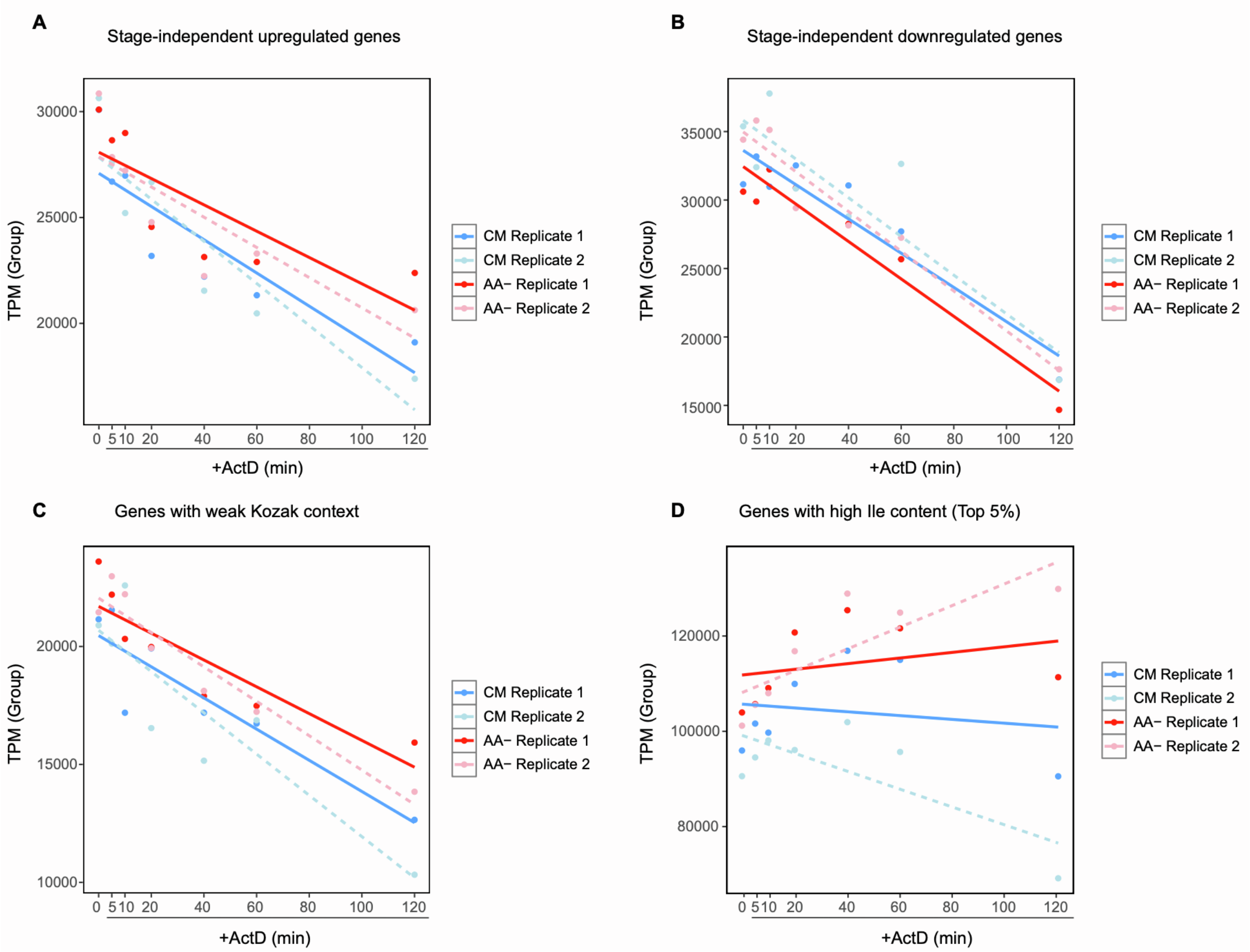
A
Stage-independent upregulated genes
TPM (Group)
+ActD (min)
B
Stage-independent downregulated genes
C
Genes with weak Kozak context
D
Genes with high Ile content (Top 5%)
CM Replicate 1
CM Replicate 2
AA− Replicate 1
AA− Replicate 2

E

Stage independent upregulated

	Gradient of change	TPM (t120/ t0)	Effect of AA-depletion
CM replicate 1	-78.5	0.63	
AA-free replicate 1	-62.1	0.74	Stabilization by 17.2%
CM replicate 2	-99.4	0.57	
AA-free replicate 2	-71.4	0.67	Stabilization by 17.8%

Stage independent downregulated

	Gradient of change	TPM (t120/ t0)	Effect of AA-depletion
CM replicate 1	-215.1	0.54	
AA-free replicate 1	-136.7	0.48	detabilization by 11.5%
CM replicate 2	-141.3	0.48	
AA-free replicate 2	-145.2	0.51	Stabilization by 7.6%

Genes with weak Kozak context

	Gradient of change	TPM (t120/ t0)	Effect of AA-depletion
CM replicate 1	-66.1	0.60	
AA-free replicate 1	-56.8	0.67	Stabilization by 12.8%
CM replicate 2	-87.5	0.49	
AA-free replicate 2	-72.9	0.65	Stabilization by 31.1%

Genes with high Ile content (Top 5%)

	Gradient of change	TPM (t120/ t0)	Effect of AA-depletion
CM replicate 1	-39.7	0.94	
AA-free replicate 1	58.8	1.07	Stabilization by 13.3%
CM replicate 2	-186.1	0.77	
AA-free replicate 2	226.1	1.28	Stabilization by 66.9%

Figure S7. Decay of transcripts with high Ile content is slowed upon AA-depletion, related to Figure 4

Plots showing the changes in summed TPM value corresponding to the group of transcripts identified as **(A)** Upregulated stage independently upon AA-depletion (n=253); **(B)** Downregulated stage independently upon AA-depletion (n=95); **(C)** having weak Kozak context (n=141) and **(D)** the top 5% in Ile usage frequency (n=263). In each plot, two replicates of a time-course experiment were shown. In each experiment, parasites were either cultured in complete RPMI or AA-free RPMI for 3 hours prior to addition of 20ug/ml actinomycin D to inhibit transcription. The changes of the transcriptome were determined with RNA samples collected at multiple timepoints over a 2-hour period. The lines represent the linear regressions for the sample sets. **(E)** A summary table showing the gradient of the regression and the t120 / t0 TPM ratio for each sample set. Weak Kozak context defined as YnnATGY (ATG= start codon).

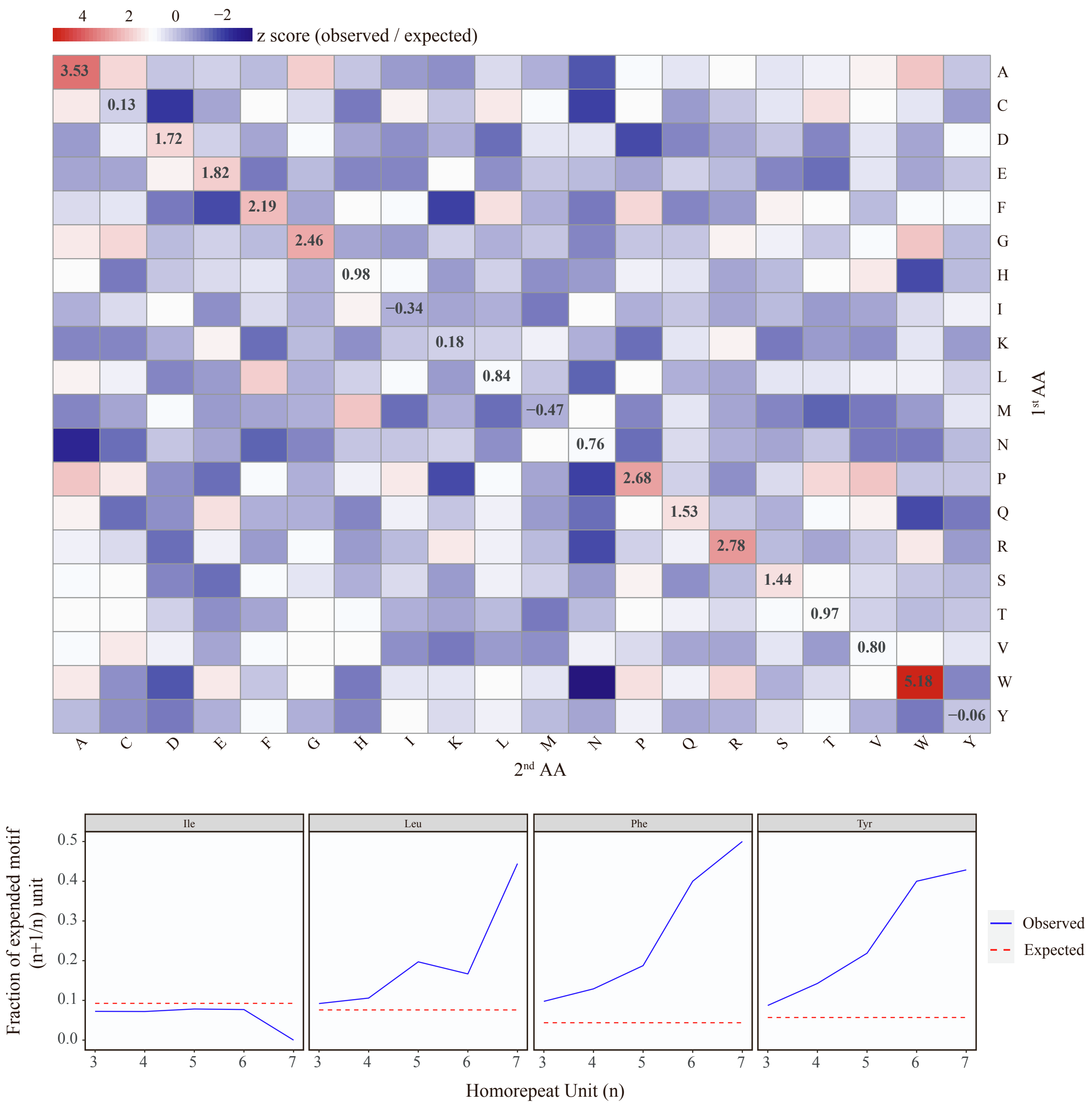

Figure S8. The expansion of Ile homorepeats is under purifying selection.

(Upper) Heatmap showing the frequency of di-amino acid motifs relative to their expected frequency. Values of (observed/expected frequency) were calculated for each di-amino acid motif, and all values were Z-transformed to generate the heatmap. (Lower) The fraction of homorepeat motifs retained with successive expansion in residue number (n) in four types of hydrophobic amino acid. The dotted lines represent the expected frequency based on the amino acid usage. Four of the most frequently used hydrophobic amino acids were analyzed.
